# Phthalate Metabolites, Consumer Habits and Health Effects

**DOI:** 10.3390/ijerph13070717

**Published:** 2016-07-15

**Authors:** Peter Wallner, Michael Kundi, Philipp Hohenblum, Sigrid Scharf, Hans-Peter Hutter

**Affiliations:** 1 Institute of Environmental Health, Center for Public Health, Medical University Vienna, Kinderspitalgasse 15, Vienna 1090, Austria; peter.wallner4@gmail.com (P.W.); michael.kundi@meduniwien.ac.at (M.K.); 2 Environmental Agency Austria, Spittelauer Lände 5, Vienna 1090, Austria; philipp.hohenblum@umweltbundesamt.at (P.H.); sigrid.scharf@umweltbundesamt.at (S.S.)

**Keywords:** human biomonitoring, urinary phthalate metabolites, consumer products, mothers, children, health effects, Austria

## Abstract

Phthalates are multifunctional chemicals used in a wide variety of consumer products. The aim of this study was to investigate whether levels of urinary phthalate metabolites in urine samples of Austrian mothers and their children were associated with consumer habits and health indicators. Within an Austrian biomonitoring survey, urine samples from 50 mother-child pairs of five communities (two-stage random stratified sampling) were analysed. The concentrations of 14 phthalate metabolites were determined, and a questionnaire was administered. Monoethyl phthalate (MEP), mono-n-butyl phthalate (MnBP), mono-isobutyl phthalate (MiBP), monobenzyl phthalate (MBzP), mono-(2-ethylhexyl) phthalate (MEHP), mono-(2-ethyl-5-hydroxyhexyl) phthalate (5OH-MEHP), mono-(2-ethyl-5-oxohexyl) phthalate (5oxo-MEHP), mono-(5-carboxy-2-ethylpentyl) phthalate (5cx-MEPP), and 3-carboxy-mono-propyl phthalate (3cx-MPP) could be quantified in the majority of samples. Significant correlations were found between the use of hair mousse, hair dye, makeup, chewing gum, polyethylene terephthalate (PET) bottles and the diethyl phthalate (DEP) metabolite MEP. With regard to health effects, significant associations of MEP in urine with headache, repeated coughing, diarrhoea, and hormonal problems were observed. MBzP was associated with repeated coughing and MEHP was associated with itching.

## 1. Introduction

Phthalates are multifunctional chemicals which are primarily used to increase the the softness and flexibility of plastics [1,2,3]. They are also used in products such as pharmaceuticals, cosmetics and personal care products, and paints [4,5].

Human exposure to phthalates is ubiquitous and can occur from food, dust, air, or contact to products which contain phthalates. After (rapid) absorption, phthalates are metabolized by hydrolysis and subsequent oxidation, and excreted mainly via urine [6].

Some phthalates, like di-(2-ethylhexyl) phthalate (DEHP), butylbenzyl phthalate (BBzP), and di-n-butyl phthalate (DnBP) have endocrine-disrupting properties [7]. Phthalates have also been associated with a variety of adverse effects, such as asthma (e.g., [8,9,10]) or effects on the cardiovascular system [11].

In this study, we investigated 50 Austrian mother-child pairs and assessed phthalate metabolites in urine. Furthermore, possible associations of phthalate levels with (i) lifestyle, consumer habits and different sources of exposure; and with (ii) health effects.

## 2. Materials and Methods

### 2.1. Recruitement

In an Austrian biomonitoring study [12], the concentrations of 14 phthalate metabolites in the urine of mother-child pairs were determined. The participants were selected based on a two-stage random stratified sampling method, taking into account the parameters gender, age, community size, and place of residence. Five groups of Austrian communities were defined according to their population size: more than one million inhabitants, 100,000–1 million inhabitants, 10,000–100,000 inhabitants, 5000–10,000 inhabitants, and less than 5000 inhabitants. With the exception of Vienna, the only Austrian city with more than one million inhabitants, a random selection of the community was performed. In each city/community (Vienna, Linz, St. Pölten, Ried, Tamsweg, Austria) 10 families were recruited by random selection from lists of telephone numbers (in total, 50 families). The first ten families in each study region that fulfilled the inclusion criteria (mother and partner: age 25–50 years; children: age 6–11; living at least one year in the same household) and consented to take part were included in the study. Severe chronic diseases in parents or children were considered exclusion criteria. The study was approved by the ethics committee of the Medical University of Vienna (045/2009).

### 2.2. Sampling and Questionnaire

An appointment was made with the families at a date and time when index persons were present for inquiry and sampling. Urine flasks were delivered on the day before the visit and participants were instructed on how to collect the morning urine. A study nurse visited the families and collected the samples. She interviewed all index persons (mother, father, and the youngest child within the range of 6 to 11 years).

Sample collection took place in February and March 2009. The questionnaire consisted of the following parts: Socio-demographic characteristics; respiratory symptoms and allergies (shortened version of the AUPHEP (Austrian Project on Health Effects of Particulates) [13] and ISAAC (The International Study of Asthma and Allergies in Childhood) questionnaire [14]), unspecific symptoms (shortened version of von Zerssen’s symptoms list [15]), allergies, regular medications, menstruation problems, fertility problems, early menarche and puberty (in children), food frequency questionnaire, use of consumer products (cosmetics, plastics, electronic devices, etc.), and living and environmental conditions.

### 2.3. Urine Analysis

In 50 mother-child pairs, morning urine samples were tested for 14 primary and secondary phthalate metabolites (see Table 1). Concentrations of creatinine were also determined. Analysis of phthalate metabolites was performed by means of liquid chromatography-electrospray ionization-tandem mass spectrometry (LC-ESI-MS/MS) according to Koch et al. (2003) [16] after enzymatic hydrolysis. Eight-hundred µL urine samples were pipetted into a centrifuge vessel and spiked with a mix of isotopically-labeled surrogates. Sodium buffer was added (0.1 M, pH 5) and the solution was shaken well. One-hundred µL of β-Glucuronidase (H. Pomatia, approx. 16.700 units per mL) was added, and the mixture was shaken again. The mixture was incubated for 2 h at 37 °C on a shaking water bath. The sample was centrifuged, and the supernatant was transferred into brown glass vial for instrumental analysis. Analysis was performed on a HP 1200 HPLC system (Hewlett Packard, Palo Alto, CA, USA) consisting of a membrane degasser, an automatic sampler, and a column heater that was coupled to a 4000 QTRAP tandem mass spectrometer (Applied Biosystems, Thermo Fisher Scientific, Waltham, MA, USA). Separation of analytes was performed using a 150 × 2 mm Luna Phenyl-Hexyl column with 3 µm pore size. Eluents were water acetonitrile 9/1 *v*/*v*, and acetonitrile/water 9/1 *v*/*v*, each modified with 1% acetic acid. The column heater was operated at 25 °C. The mass spectrometer source was operated in the negative ESI (Electrospray Ionization) mode. Quantitation was performed in the multiple reaction monitoring (MRM) mode.

Limits of quantitation (LOQ) were 1.0 µg/L for monoethyl phthalate (MEP), 0.8 µg/L for monobenzyl phthalate (MBzP), 0.6 µg/L for mono-cyclohexyl phthalate (MCHP), 1.0 µg/L for mono-isobutyl phthalate (MiBP), 0.8 µg/L for mono-n-butyl phthalate (MnBP), 1.0 µg/L for 3-carboxy-mono-propyl phthalate (3cx-MPP) and mono-n-pentyl phthalate (MnPEP), 0.81 µg/L for mono-(2-ethylhexyl) phthalate (MEHP), 0.24 µg/L for mono-(2-ethyl-5-hydroxyhexyl) phthalate (5OH-MEHP), 1.3 µg/L for mono-(5-carboxy-2-ethylpentyl) phthalate (5cx-MEPP), 0.5 µg/L for mono-(2-ethyl-5-oxohexyl) phthalate (5oxo-MEHP), 0.6 µg/L for mono-n-octyl phthalate (MnOP), 0.8 µg/L for mono-isononyl phthalate (MiNP), and 1.0 for mono-isodecyl phthalate (MiDP). Limits of detection (LOD) were ≤0.5 µg/L for all compounds.

For validation of the analytical methods, interlaboratory test samples were analysed. The results matched the expected results within the accuracy limits. The between-day imprecision, expressed as relative standard deviation, ranged between 3.8% and 8.6%, the mean relative recoveries were between 77.3% and 128%.

Due to repeated measurements, material was in some cases too little, so all 14 metabolites could only be analysed in 92 samples and MEHP in 99 samples.

### 2.4. Statistical Analysis

Creatinine-corrected metabolite concentrations were square-root transformed to obtain a less-skewed distribution. Values below the LOD were arbitrarily set to half the LOD. Deviation from normality was assessed by Kolmogorov-Smirnov tests with Lilliefors’ corrected *p*-values. Data for frequency of use of consumer and food products were analysed for correlation with phthalate metabolites by Spearman rank-correlation. Presence of symptoms in relation to metabolite concentrations was analysed by logistic regression with age (and gender in children) as covariates. Odds ratios (OR) and 95% confidence intervals (CI) were computed for an increase of one standard deviation of the metabolite. Differences between maternal and child metabolite concentrations were assessed by paired *t*-tests and correlations by Spearman coefficients. For all analyses, *p*-values below 0.05 were considered significant.

## 3. Results

Fifty mother-child pairs were available for analysis. The average age of the mothers was 38 years (±5.2 SD). Of the 50 children studied, the average age was 8 years (±1.6 SD), and 28 were boys.

Out of the 14 phthalate metabolites investigated, 9 were found in concentrations above the LOQ (creatinine-corrected values, see Table 1 and Table 2) in the majority of samples. MEP, MnBP, MiBP, MBzP, MEHP, 5OH-MEHP, 5oxo-MEHP, 5cx-MEPP, and 3cx-MPP could be quantified in the majority of the samples. MCHP and MnOP could be detected in only one sample. MnPeP, MiNP, and MiDP could not be detected.

With the exception of MEP, concentrations in samples from children were higher than in those of mothers. Creatinine-unadjusted MEHP values were also slightly higher in mothers.

Significant correlations in mothers were found between the use of some consumer products (hair mousse: R = 0.40, hair dye: R = 0.44, makeup: R = 0.46, chewing gum: R = 0.37, PET bottles: R = 0.46) and the diethyl phthalate metabolite MEP (Table 3).

Due to the fact that children did not use cosmetics products regularly, we only assessed the relationship between chewing gum and PET bottles use with phthalate metabolites in children: R = 0.26 (not significant) for MEP, 0.46 (*p* < 0.01) for MBzP and chewing gum use; R = 0.33 (*p* < 0.05) for MEP and use of PET bottles.

Highly significant Spearman correlations were found between maternal and child urinary metabolite concentrations, except for 5OH-MEHP. The largest correlations were found for MEP (R = 0.64) and MnBP (R = 0.52).

Significant associations with symptoms and health problems were observed, especially with concentrations of MEP. This was the case for “headache” (in the last 3 months) in children (OR 1.38, 95% CI: 1.15–1.66), “repeated coughing” (in the last 3 months) in children (OR 2.38, 95% CI: 1.06–5.33), “diarrhoea” (in the last 3 months) in mothers (OR 1.44, 95% CI: 1.18–1.76) and children (OR 4.48, 95% CI: 1.31–15.26), and “hormonal problems” in mothers (OR 1.47, 95% CI: 1.10–1.98). MBzP was associated with cough in mothers (OR 1.52, 95% CI: 1.13–2.04) and children (OR 1.99, 95% CI: 1.23–3.23), MEHP with itching (in the last 3 months) in mothers (OR 1.93, 95% CI: 1.10–3.31). For further details see Table 4 and Table 5.

## 4. Discussion

Considering the data of the DEMOCOPHES (Demonstration of a study to Coordinate and Perform. Human biomonitoring on a. European Scale) study, our results were rather low compared to other Western European Countries [17]. Furthermore, data from Eastern European countries [18] also showed higher phthalate concentrations. On the other hand, our results were comparable to findings in Denmark (sampling period 2011) [19] and from another Austrian study (sampling period 2010/2012) [6]. Similar to other studies, children showed higher values of phthalates metabolites [3,20,21,22].

It is known that diethyl phthalate is widely used in cosmetic products. In addition, make-up, hair dye, and hair mousse particularly are amongst the most frequently used cosmetics. Since DEP is added to cosmetic products, the described correlations with its metabolite MEP are plausible. The frequent use of these cosmetic products leads to a higher internal burden due to absorption through the skin.

Frequently enjoying chewing gum also showed a correlation with the MEP concentration in urine. On one hand, the gum itself might contain phthalates like DEP (e.g., [23]). On the other hand, it is also conceivable that plasticizers could pass into the chewing gum from the packaging. In a study on Swedish mother-child couples, mothers who consumed chewing gum several times a week also showed higher MEP levels [3]. High consumption of chewing gum was related to higher MEP levels in mothers in the large European DEMOCOPHES study, too [17].

Polyethylene terephthalate (PET) is a plastic from which beverage bottles are produced, for example, as well as other packaging for food. Plastic bottles are now increasingly used in the beverage industry in place of glass bottles. Plasticizers are not used in the production of PET bottles [24]. Nonetheless, phthalates could still be detected in bottles [25,26] and in soft drinks and mineral water (e.g., [26,27]), which highlights the importance of the quality of PET raw material used for the production [25,26]. DEP could be found in water from plastic bottles [28,29]. It is therefore not implausible that significantly higher MEP concentrations are found in the urine of people who drink more frequently from PET bottles.

The link between exposure to phthalates and symptoms of headache, itching, and diarrhoea was, to the best of our knowledge, not observed in any environmental health study to date. However, it is known from the literature that at high doses headache and diarrhoea may occur [30,31] (workplace, animal testing, etc.). It should also be noted that headache is often reported in association with exposure to indoor air pollutants [32]. It might be possible that phthalates have made some contribution to the occurrence of headaches in these cases or they might serve as indicators of indoor air pollution.

Dryness of the skin/mucous membranes—frequently associated with itching—is recognised as one of the problems caused by age-related postmenopausal oestrogen deficiency. Therapeutically, oestrogen-containing creams, etc. are indicated. In this context, oestrogens counteract the itching. A possible explanation of why in our study increased itching occurs where higher phthalate concentrations in urine are present could be that it is due to their acting in competition with the natural hormones. The effect (occurrence of itching) could speculatively be explained in that phthalates bind to oestrogen receptors [33], leaving a smaller number of receptors available for the endogenous oestrogens. Due to the limitations of a cross sectional study, this association is not necessarily causal and could have alternative explanations: For example, a person could already have experienced itching and therefore used more (dermatological) creams that contain phthalates.

The association between the DEHP metabolite MEHP and itching might also be explained by the known association between DEHP and allergies demonstrated in epidemiological studies [34] and in case studies [35]. In mice, DEHP enhanced atopic dermatitis-like skin lesions [36]. However, an association of other DEHP metabolites with itching was not found in our study. This might be due to different half-life of the phthalate metabolites.

In interior rooms, there are usually numerous phthalate-containing materials which can release phthalates into both the air in the room and into house dust. Several studies have addressed the effects on lung function and inflammatory effects on the respiratory tract caused by the inhalation of phthalates. Monoethyl phthalate (MEP) was associated with lower FVC (Forced Vital Capacity) and FEV1 (Forced Expiratory Volume at 1 s) values in male participants of the U.S. National Health and Nutrition Examination Survey (NHANES III) survey [37]. In the NHANES 2005–2006 survey, the benzylbutyl phthalate metabolite MBzP was associated with current allergic symptoms in adults [38]. In the Austrian school study “Air and children” there were significant inverse correlations between benzylbutyl phthalate (in house dust) and the lung function (reduction of MEF75) of school children [39]. It is well known that phthalates in school buildings as well as day care centres can be found in higher concentrations [40,41]. The observations in our study—the correlation of repeated coughing with the metabolites MEP and MBzP—seem plausible, and might be seen as further evidence of the effects of phthalates on the respiratory tract.

Adverse effects of phthalates on endocrine and reproductive functions in humans have been demonstrated in many investigations. We found a significant association between elevated MEP concentrations in the urine of mothers and an increased percentage of hormonal problems. This is based mainly on the evaluation of answers to the question “Did you have hormonal problems at any time (e.g., a prolonged period of time until you became pregnant)”. In this instance, it is assumed that the test subjects answered the question mainly in respect of problems regarding a delayed pregnancy. Other hormonal abnormalities were not disclosed (with one exception of a case of early menarche).

In this study, investigations of the secondary metabolites of the phthalates DiNP and DiBP have not yet been performed. Because of their importance, these metabolites will be included in future measurements.

## 5. Conclusions

In this Austrian study on 50 mother-child pairs, the concentrations of phthalate metabolites were rather low. Significant correlations were found between the use of some cosmetics, chewing gum, and PET bottles and the diethyl phthalate metabolite MEP.

With regard to health effects, significant associations of MEP in urine with headache, repeated coughing, diarrhoea, and hormonal problems were observed. The benzylbutyl phthalate metabolite MBzP was associated with repeated coughing, MEHP with itching. As this study was cross sectional, the associations found do not necessarily indicate causation. Furthermore, the answers to the questions regarding health symptoms can be subject to recall bias and probably misclassification bias. Excretion follows a diurnal pattern and thus, spot samples may vary significantly in their composition. As recommended by COPHES, first morning void samples were taken to guarantee that diurnal variation does not affect the results.

As some of these associations have not been reported previously, confirmation is required in future trials.

## Figures and Tables

**Table 1:** Creatinine-adjusted concentrations of phthalate metabolites in urine samples of mothers (in µg/g creatinine) (*secondary metabolites in italics*).

Phthalate Metabolite	*n*	>LOQ	Min	Max	Median	P95
Monoethyl phthalate (MEP)	48	48	3.9	437	35	165
Monobenzyl phthalate (MBzP)	48	37	<LOD	6.2	1.3	3.6
Mono-cyclohexyl phthalate (MCHP)	48	0	<LOD	<LOD	-	-
Mono-isobutyl phthalate (MiBP)	48	43	<LOD	20	8	15
Mono-n-butyl phthalate (MnBP)	48	48	1.1	30	5.6	18
*3-carboxy-mono-propyl phthalate (3cx-MPP)*	48	46	<LOQ	15	2.8	11
Mono-n-pentyl phthalate (MnPeP)	48	0	<LOD	<LOD	-	-
Mono-(2-ethylhexyl) phthalate (MEHP)	50	32	<LOD	39	0.8	5.3
*Mono-(2-ethyl-5-hydroxyhexyl) phthalate (5OH-MEHP)*	50	49	<LOQ	183	2.3	10
*Mono-(5-carboxy-2-ethylpentyl) phthalate (5cx-MEPP)*	50	50	3.1	1057	12	95
*Mono-(2-ethyl-5-oxohexyl) phthalate (5oxo-MEHP)*	48	45	<LOQ	87	1.5	6.1
Mono-n-octyl phthalate (MnOP)	48	0	<LOD	<LOQ	-	-
Mono-isononyl phthalate (MiNP)	48	0	<LOD	<LOD	-	-
Mono-isodecyl phthalate (MiDP)	48	0	<LOD	<LOD	-	-

LOD: Limit of Detection; LOQ: Limit of Quantitation; P95, 95th percentile.

**Table 2:** Creatinine-adjusted concentrations of phthalate metabolites in urine samples of children (in µg/g creatinine) (*secondary metabolites in italics*).

Phthalate Metabolite	*n*	>LOQ	Min	Max	Median	P95
Monoethyl phthalate (MEP)	44	44	3.6	340	25	212
Monobenzyl phthalate (MBzP)	44	41	<LOD	23	2.0	9.3
Mono-cyclohexyl phthalate (MCHP)	44	0	<LOD	<LOQ	-	-
Mono-isobutyl phthalate (MiBP)	44	42	<LOD	87	10	36
Mono-n-butyl phthalate (MnBP)	44	44	1.1	42	7.9	25
*3-carboxy-mono-propyl phthalate (3cx-MPP)*	44	44	1.8	251	5.9	20
Mono-n-pentyl phthalate (MnPeP)	44	0	<LOD	<LOD	-	-
Mono-(2-ethylhexyl) phthalate (MEHP)	49	44	<LOD	11.1	0.9	7.9
*Mono-(2-ethyl-5-hydroxyhexyl) phthalate (5OH-MEHP)*	50	50	0.7	27	4.4	10
*Mono-(5-carboxy-2-ethylpentyl) phthalate* *(5cx-MEPP)*	50	50	6.9	120	28	82
*Mono-(2-ethyl-5-oxohexyl) phthalate* *(5oxo-MEHP)*	44	32	<LOQ	12	2.9	6.2
Mono-n-octyl phthalate (MnOP)	44	0	<LOD	<LOD	-	-
Mono-isononyl phthalate (MiNP)	44	0	<LOD	<LOD	-	-
Mono-isodecyl phthalate (MiDP)	44	0	<LOD	<LOD	-	-

LOD: Limit Of Detection; LOQ: Limit of Quantitation; P95, 95th percentile.

**Table 3:** Spearman correlation coefficients (* *p* < 0.05) of phthalate metabolites and intensity of use of some consumer products in mothers.

Metabolites	Hair Dye	Make Up	Perfume	Hair Mousse	Skin Lotion	Nail Polish	Chewing Gum	PET Bottles
MEP	0.44 *	0.46 *	−0.21	0.40 *	0.29	0.25	0.37 *	0.46 *
MBzP	−0.02	−0.27	−0.06	−0.26	0.09	0.30 *	0.30 *	0.07
MiBP	−0.01	−0.06	−0.12	−0.12	−0.14	−0.11	−0.19	0.23
MnBP	−0.10	−0.01	−0.28	0.36 *	0.10	0.01	0.05	0.08
3cx-MPP	−0.25	−0.03	0.20	−0.23	−0.11	0.19	−0.12	−0.19
MEHP	0.01	−0.13	−0.03	−0.22	−0.10	−0.01	−0.15	−0.02
5OH-MEHP	0.26	0.46 *	−0.21	0.40 *	−0.29	−0.25	0.16	0.06
5cx-MEPP	−0.17	0.02	0.04	0.31 *	−0.05	0.05	0.19	−0.09
5oxo-MEHP	−0.13	−0.16	0.04	−0.27	−0.03	0.16	−0.06	−0.04

**Table 4:** Results of logistic regression of symptoms during the last 3 months in children on phthalate metabolites. Odds ratios and 95% confidence intervals for an increase of one standard deviation of the metabolite.

Metabolite	Repeated Cough	Headache	Unusual Tiredness	Vertigo	Diarrhea	Nausea	Vomiting	Itching
MEP	2.38 (1.06–5.33)	1.38 (1.15–1.66)	1.21 (0.66–2.23)	0.17 (0.00–7.41)	4.48 (1.31–15.26)	2.17 (1.03–4.57)	1.92 (0.94–3.92)	1.24 (0.68–2.29)
MBzP	1.99 (1.23–3.23)	0.94 (0.50–1.77)	0.71 (0.35–1.46)	0.73 (0.13–4.04)	1.24 (0.67–2.31)	0.75 (0.35–1.60)	0.51 (0.16–1.70)	1.30 (0.70–2.42)
MiBP	1.08 (0.54–2.18)	1.26 (0.68–2.35)	0.91 (0.48–1.73)	0.66 (0.12–3.50)	0.75 (0.38–1.50)	1.09 (0.56–2.11)	1.05 (0.45–2.47)	1.10 (0.59–2.03)
MnBP	1.25 (0.64–2.47)	1.68 (0.87–3.27)	0.74 (0.37–1.48)	0.40 (0.05–3.34)	1.17 (0.63–2.17)	1.59 (0.82–3.08)	1.47 (0.68–3.18)	1.37 (0.73–2.56)
3cxMPP	1.12 (0.59–2.11)	1.01 (0.55–1.87)	0.16 (0.02–1.51)	0.00 (0.00–8.31)	0.83 (0.38–1.83)	0.97 (0.48–1.94)	1.08 (0.50–2.33)	1.41 (0.68–2.93)
MEHP	0.75 (0.36–1.57)	1.30 (0.72–2.33)	1.15 (0.64–2.05)	1.31 (0.39–4.48)	0.70 (0.35–1.40)	1.21 (0.66–2.21)	1.23 (0.58–2.58)	1.82 (0.95–3.47)
OHMEHP	0.80 (0.39–1.64)	1.65 (0.87–3.15)	0.74 (0.39–1.41)	0.23 (0.02–2.25)	0.93 (0.51–1.72)	1.21 (0.66–2.22)	1.17 (0.55–2.49)	1.31 (0.72–2.37)
5cxMEPP	0.83 (0.42–1.63)	1.56 (0.86–2.85)	0.68 (0.37–1.28)	0.65 (0.12–3.40)	0.87 (0.47–1.61)	0.75 (0.39–1.46)	0.91 (0.40–2.08)	1.16 (0.64–2.08)
5oxoMEHP	1.15 (0.57–2.32)	1.54 (0.80–2.96)	0.88 (0.46–1.67)	0.58 (0.12–2.87)	0.99 (0.53–1.86)	1.35 (0.69–2.65)	1.70 (0.73–3.96)	1.43 (0.75–2.71)

**Table 5:** Results of logistic regression of symptoms during the last 3 months in mothers on phthalate metabolites. Odds ratios and 95% confidence intervals for an increase of one standard deviation of the metabolite.

Metabolite	Repeated Cough	Headache	Unusual Tiredness	Vertigo	Diarrhea	Nausea	Vomiting	Itching
MEP	1.12 (0.56–2.24)	1.19 (0.62–2.32)	0.54 (0.28–1.04)	0.66 (0.32–1.36)	1.44 (1.18–1.76)	1.74 (0.92–3.32)	1.27 (0.46–3.48)	0.52 (0.23–1.19)
MBzP	1.52 (1.13–2.04)	1.09 (0.59–2.02)	1.47 (0.75–2.85)	0.94 (0.52–1.71)	0.82 (0.44–1.52)	1.12 (0.61–2.04)	0.61 (0.19–1.98)	1.66 (0.86–3.20)
MiBP	0.69 (0.35–1.38)	1.27 (0.70–2.33)	0.91 (0.47–1.75)	0.65 (0.35–1.19)	0.72 (0.40–1.33)	0.92 (0.51–1.67)	0.51 (0.20–1.30)	1.03 (0.56–1.88)
MnBP	1.32 (0.66–2.65)	1.40 (0.71–2.78)	1.01 (0.53–1.92)	0.98 (0.54–1.78)	1.46 (0.79–2.72)	1.28 (0.71–2.32)	0.88 (0.25–3.07)	1.66 (0.88–3.13)
3cx-MPP	1.38 (0.71–2.71)	0.82 (0.45–1.49)	0.98 (0.52–1.86)	0.86 (0.46–1.60)	0.95 (0.51–1.77)	0.88 (0.47–1.63)	0.66 (0.15–2.96)	0.78 (0.41–1.49)
MEHP	1.43 (0.76–2.67)	1.17 (0.60–2.26)	0.92 (0.51–1.65)	0.79 (0.39–1.59)	1.25 (0.70–2.25)	1.09 (0.62–1.93)	0.08 (0.01–1.09)	1.93 (1.10–3.31)
5OH-MEHP	1.93 (0.73–5.14)	1.09 (0.57–2.10)	1.13 (0.54–2.37)	0.70 (0.26–1.89)	2.12 (0.63–7.18)	1.23 (0.68–2.21)	0.01 (0.00–1.40)	1.44 (0.70–2.95)
5cx-MEPP	3.39 (0.89–12.94)	0.96 (0.54–1.72)	1.19 (0.54–2.65)	0.60 (0.19–1.89)	1.73 (0.73–4.11)	1.10 (0.63–1.94)	0.01 (0.00–15.36)	1.44 (0.72–2.89)
5oxo-MEHP	2.28 (0.71–7.28)	0.96 (0.53–1.75)	1.14 (0.53–2.48)	0.62 (0.19–1.97)	2.04 (0.67–6.20)	1.28 (0.69–2.37)	0.00 (0.00–0.67)	1.64 (0.69–3.92)

## Abbreviations

The following abbreviations are used in this manuscript:

3-cx-MPP	3-carboxy-mono-propyl phthalate: secondary metabolite of di-n-octyl phthalate, di-n-butyl phthalate and/or di-isononyl phthalate
5-cx-MEPP	Mono-(5-carboxy-2-ethylpentyl)phthalate: secondary metabolite of di-(2-ethylhexyl) phthalate
5OH-MEHP	Mono-(2-ethyl-5-hydroxyhexyl) phthalate: secondary metabolite of di-(2-ethylhexyl) phthalate
5oxo-MEHP	Mono-(2-ethyl-5-oxohexyl) phthalate: secondary metabolite of di-(2-ethylhexyl) phthalate
BPA	Bisphenol-A
LC-ESI-MS/MS	Liquid chromatography-electrospray ionization-tandem mass spectrometry
LOD	Limit of detection
LOQ	Limit of quantification
MBzP	Mono-benzyl phthalate: primary metabolite of butylbenzyl phthalate
MCHP	Mono-cyclohexyl phthalate: primary metabolite of dicyclohexyl phthalate
MEHP	Mono-(2-ethylhexyl) phthalate: primary metabolite of di(2-ethylhexyl)phthalate
MEP	Monoethyl phthalate: primary metabolite of diethyl phthalate
MiBP	Mono-isobutyl phthalate: primary metabolite of di-isobutyl phthalate
MiDP	Mono-isodecyl phthalate: primary metabolite of di-isodecyl phthalate
MiNP	Mono-isononyl phthalate: primary metabolite of di-isononyl phthalate
MnBP	Mono-n-butyl phthalate: primary metabolite of di-n-butyl phthalate
MnOP	Mono-n-octyl phthalate: primary metabolite of dioctyl phthalate
MnPeP	Mono-n-pentyl phthalate: primary metabolite of di-n-pentyl phthalate
OR	Odds Ratio

## References

[1] NRC (National Research Council) (2008). Phthalates and Cumulative Risk Assessment. The Task Ahead.

[2] Danish EPA (2013). Phthalate Strategy.

[3] Larsson K., Björklund K.L., Palm B., Wennberg M., Kaj L., Lindh C.H., Jönnson B.A.G., Berglund M. (2014). Exposure determinants of phthalates, parabens, bisphenol A and triclosan in Swedish mothers and their children. Environ. Int..

[4] CDC (Centers for Disease Control and Prevention) National Biomonitoring Program. Factsheet Phthalates, 2015. http://www.cdc.gov/biomonitoring/phthalates_factsheet.html.

[5] Wittassek M., Koch H.M., Angerer J., Brüning T. (2011). Assessing exposure to phthalates—The human biomonitoring approach. Mol. Nutr. Food Res..

[6] Hartmann C., Uhl M., Weiss S., Koch H.M., Scharf S., König J. (2015). Human biomonitoring of phthalate exposure in Austrian children and adults and cumulative risk assessment. Int. J. Hyg. Environ. Health.

[7] Bergman A., Heindel J.J., Jobling S., Kidd K.A., Zoeller R.T., WHO/UNEP (2013). State of the Science of Endocrine Disrupting Chemicals—2012.

[8] Bornehag C.G., Sundell J., Weschler C.J., Sigsgaard T., Lundgren B., Hasselgren M., Hägerhed-Engman L. (2004). The association between asthma and allergic symptoms in children and phthalates in house dust: A nested case-control study. Environ. Health Perspect..

[9] Bertelsen R.J., Carlsen K.C., Calafat A.M., Hoppin J.A., Håland G., Mowinckel P., Carlsen K.H., Løvik M. (2013). Urinary biomarkers for phthalates associated with asthma in Norwegian children. Environ. Health Perspect..

[10] Ait Bamai Y., Shibata E., Saito I., Araki A., Kanazawa A., Morimoto K., Nakayama K., Tanaka M., Takigawa T., Yoshimura T. (2014). Exposure to house dust phthalates in relation to asthma and allergies in both children and adults. Sci. Total Environ..

[11] Posnack N.G. (2014). The adverse cardiac effects of Di(2-ethylhexyl)phthalate and Bisphenol A. Cardiovasc. Toxicol..

[12] Hohenblum P., Steinbichl P., Raffesberg W., Weiss S., Moche W., Vallant B., Scharf S., Haluza D., Moshammer H., Kundi M. (2012). Pollution gets personal! A first population-based human biomonitoring study in Austria. Int. J. Hyg. Environ. Health.

[13] Asher M.I., Keil U., Anderson H.R., Beasley R., Crane J., Martinez F., Mitchell E.A., Pearce N., Sibbald B., Stewart A.W. (1995). International Study of Asthma and Allergies in Childhood (ISAAC): Rationale and methods. Eur. Respir. J..

[14] Hauck H., Berner A., Frischer T., Gomiscek B., Kundi M., Neuberger M., Puxbaum H., Preining O. (2004). AUPHEP—Austrian project on health effects of particulates—General overview. Atmos. Environ..

[15] Zerssen D.V., Koeller D.M. (1976). Die Befindlichkeitsskala.

[16] Koch H.M., Gonzalez-Reche L.M., Angerer J. (2003). On-line clean-up by multidimensional liquid-chromatography electrospray ionization tandem massspectrometry for high throughput quantification of primary and secondary phthalate metabolites in human urine. J. Chromatogr. B.

[17] Den Hond E., Govarts E., Willems H., Smolders R., Casteleyn L., Kolossa-Gehring M., Schwedler G., Seiwert M., Fiddicke U., Castaño A. (2015). First steps toward harmonized human biomonitoring in Europe: Demonstration project to perform human biomonitoring on a European scale. Environ. Health Perspect..

[18] Cerná M., Maly M., Rudnai P., Kozepesy S., Miklós Náray Halzlova K., Jajcaj M., Grafnetterová A., Krsková A., Antošová D., Forysová K. (2015). Case study: Possible differences in phthalates exposure among the Czech, Hungarian, and Slovak populations identified based on the DEMOCOPHES pilot study results. Environ. Res..

[19] Frederiksen H., Nielsen J.K., Mørck T.A., Hansen P.W., Jensen J.F., Nielsen O., Andersson A.M., Knudsen L.E. (2013). Urinary excretion of phthalate metabolites, phenols and parabens in rural and urban Danish mother-child pairs. Int. J. Hyg. Environ. Health.

[20] Cutanda F., Koch H.M., Esteban M., Sánchez J., Angerer J., Castaño A. (2015). Urinary levels of eight phthalate metabolites and bisphenol A in mother-child pairs from two Spanish locations. Int. J. Hyg. Environ. Health.

[21] Koch H.M., Preuss R., Drexler H., Angerer J. (2005). Exposure of nursery school children and their parents and teachers to di-n-butylphthalate and butylbenzylphthalate. Int. Arch. Occup. Environ. Health.

[22] Silva M.J., Barr D.B., Reidy J.A., Malek N.A., Hodge C.C., Caudill S.P., Brock J.W., Needham L.L., Calafat A.M. (2004). Urinary levels of seven phthalate metabolites in the U.S. population from the National Health and Nutrition Examination Survey (NHANES) 1999–2000. Environ. Health Perspect..

[23] Abdel-Malik M.M., Vishwan A., Orama A.M. (2003). Non-Stick Chewing Gum Base. U.S. Patent.

[24] BfR (Bundesinstitut für Risikobewertung) Selected Questions and Answers on PET Bottles, 2015. http://www.bfr.bund.de/en/selected_questions_and_answers_on_pet_bottles-60846.html.

[25] Sax L. (2010). Polyethylene terephthalate may yield endocrine disruptors. Environ. Health Perspect..

[26] Keresztes S., Tatár E., Czégény Z., Záray G., Mihucz V.G. (2013). Study on the leaching of phthalates from polyethylene terephthalate bottles into mineral water. Sci. Total Environ..

[27] Bošnir J., Puntarić D., Galić A., Škes I., Dijanić T., Klarić M., Grgić M., Čurković M., Šmit Z. (2007). Migration of phthalates from plastic containers into soft drinks and mineral water. Food Technol. Biotechnol..

[28] Al-Saleh I., Shinwari N., Alsabbaheen A. (2011). Phthalates residues in plastic bottled waters. J. Toxicol. Sci..

[29] Casajuana N., Lacorte S. (2003). Presence and release of phthalic esters and other endocrine disrupting compounds in drinking water. Chromatographia.

[30] Krauskopf L.G. (1973). Studies on the toxicity of phthalates via ingestion. Environ. Health Perspect..

[31] New Jersey Department of Health and Senior Services Right to Know. Hazardous Substances Fact Sheet. Diethyl Phthalate, 2010. http://nj.gov/health/eoh/rtkweb/documents/fs/0707.pdf.

[32] EPA (U.S. Environmental Protection Agency) An Introduction to Indoor Air Quality, 2015. http://www2.epa.gov/indoor-air-quality-iaq/introduction-indoor-air-quality.

[33] Ohashi A., Kotera H., Hori H., Hibiya M., Watanabe K., Murakami K., Hasegawa M., Tomita M., Hiki Y., Sugiyama S. (2005). Evaluation of endocrine disrupting activity of plasticizers in polyvinyl chloride tubes by estrogen receptor alpha binding assay. J. Artif. Organs.

[34] Jaakkola J.J., Knight T.L. (2008). The role of exposure to phthalates from polyvinylchloride products in the development of asthma and allergies: A systematic review and meta-analysis. Environ. Health Perspect..

[35] Sugiura K., Sugiura M., Hayakawa R., Shamoto M., Sasaki K. (2002). A case of contact urticaria syndrome due to di(2-ethylhexyl) phthalate (DOP) in work clothes. Contact Dermat..

[36] Takano H., Yanagisawa R., Inoue K., Ichinose T., Sadakane K., Yoshikawa T. (2006). Di-(2-ethylhexyl)phthahalte enhances atopic dermatitis-like skin lesion in mice. Envion. Health Perspect..

[37] Hoppin J.A., Ulmer R., London S.J. (2004). Phthalate exposure and pulmonary function. Environ. Health Perspect..

[38] Hoppin J.A., Jaramillo R., London S.J., Bertelsen R.J., Salo P.M., Sandler D.P., Zeldin D.C. (2013). Phthalate exposure and allergy in the U.S. population: Results from NHANES 2005–2006. Environ. Health Perspect..

[39] Wallner P., Kundi M., Moshammer H., Piegler K., Hohenblum P., Scharf S., Fröhlich M., Damberger B., Tappler P., Hutter H.P. (2012). Indoor air in schools and lung function of Austrian school children. J. Environ. Monit..

[40] Raffy G., Mercier F., Blanchard O., Derbez M., Dassonville C., Bonvallot N., Glorennec P., Le Bot B. (2016). Semi-volatile organic compounds in the air and dust of 30 French schools: A pilot study. Indoor Air.

[41] Fromme H., Lahrz T., Kraft M., Fembacher L., Burghardt R., Sievering S., Dietrich S., Völkel W. (2013). The occurrence of plasticisers (phthalates) in communal facilities under special consideration of results from LUPE 3. Gesundheitswesen.

